# Supplementary material for: Deep learning for predicting prognostic consensus molecular subtypes in cervical cancer from histology images
Source: NPJ Precis Oncol. 2025 Jan 11;9:11. doi: 10.1038/s41698-024-00778-5 (PMC11724963; doi:10.1038/s41698-024-00778-5)
Supplement: Supplementary file 1 — Supplementary information - revised [file 41698_2024_778_MOESM1_ESM.docx]

**Supplementary Tables:**

**Supplementary Table 1:** C-Indexes and DeLong test results of Molecular-CMS and Digital-CMS classifications on TCGA-CESC and Oslo-CSCC cohorts for DFS.

| DFS | C-Index | | DeLong Test | | |
| --- | --- | --- | --- | --- | --- |
|  | Molecular-CMS | Digital-CMS | DeLong Z | p value | 95% CI |
| TCGA-CESC as an external cohort | 0.62 | 0.63 | 0.17 | 0.87 | [-0.11, 0.13] |
| Oslo-CSCC as an external cohort | 0.55 | 0.57 | 0.39 | 0.7 | [-0.05, 0.07] |

**Supplementary Table 2:** C-Indexes and DeLong test results of Molecular-CMS and Digital-CMS classifications on TCGA-CESC and Oslo-CSCC cohorts for DSS.

| DSS | C-Index | | DeLong Test | | |
| --- | --- | --- | --- | --- | --- |
|  | Molecular-CMS | Digital-CMS | DeLong Z | p value | 95% CI |
| TCGA-CESC as an external cohort | 0.62 | 0.64 | 0.43 | 0.67 | [-0.08, 0.13] |
| Oslo-CSCC as an external cohort | 0.54 | 0.57 | 0.65 | 0.52 | [-0.05, 0.10] |

**Supplementary Data Titles:**

**Supplementary Data 1:** Multivariate analysis of risk factors on Uganda-CSCC cohort on overall survival.

**Supplementary Data 2:** Clinical characteristics of TCGA-CESC cohort. Digital-CMS classification was derived from Digital-CMS score using cutoff value of 0.2167, selected on the discovery cohort using Youden's J statistic.

**Supplementary Data 3:** Clinical characteristics of Oslo-CSCC cohort. Digital-CMS classification was derived from Digital-CMS score using cutoff value of 0.381, selected on the discovery cohort using Youden's J statistic.

**Supplementary Data 4:** Statistical results of differences between C1 and C2 representative regions on cellular features identified with HoverNet-Pannuke, on TCGA-CESC cohort.

**Supplementary Data 5:** Statistical results of differences between C1 and C2 representative regions on cellular features identified with HoverNet-Pannuke, on Oslo-CSCC cohort.

**Supplementary Data 6:** Statistical results of differences between C1 and C2 representative regions on cellular features identified with HoverNet-Pannuke, on Uganda-CSCC cohort.

**Supplementary Data 7:** Statistical results of differences between C1 and C2 representative regions on cellular features identified with AugHoverNet-Conic, on TCGA-CESC cohort.

**Supplementary Data 8:** Statistical results of differences between C1 and C2 representative regions on cellular features identified with AugHoverNet-Conic, on Oslo-CSCC cohort.

**Supplementary Data 9:** Statistical results of differences between C1 and C2 representative regions on cellular features identified with AugHoverNet-Conic, on Uganda-CSCC cohort.

**Supplementary Data 10:** Statistical results of differences between C1 and C2 tumours (WSI-level) on cellular features identified with HoverNet-Pannuke, on TCGA-CESC cohort.

**Supplementary Data 11:** Statistical results of differences between C1 and C2 tumours (WSI-level) on cellular features identified with HoverNet-Pannuke, on Oslo-CSCC cohort.

**Supplementary Data 12:** Statistical results of differences between C1 and C2 tumours (WSI-level) on cellular features identified with HoverNet-Pannuke, on Uganda-CSCC cohort.

**Supplementary Data 13:** Statistical results of differences between C1 and C2 tumours (WSI-level) on cellular features identified with AugHoverNet-Conic, on TCGA-CESC cohort.

**Supplementary Data 14:** Statistical results of differences between C1 and C2 tumours (WSI-level) on cellular features identified with AugHoverNet-Conic, on Oslo-CSCC cohort.

**Supplementary Data 15:** Statistical results of differences between C1 and C2 tumours (WSI-level) on cellular features identified with AugHoverNet-Conic, on Uganda-CSCC cohort.
